# Evaluating Bacteriophage Impact on *Vibrio* Composition in the Gut of Broad‐Nosed Pipefish (*Syngnathus typhle*)

**DOI:** 10.1111/1758-2229.70125

**Published:** 2025-08-07

**Authors:** Jamie Parker, Silke‐Mareike Marten, Jelena Rajkov, Franziska I. Theising, Arseny Dubin, Olivia Roth

**Affiliations:** ^1^ Marine Evolutionary Biology Zoological Institute, Kiel University Kiel Germany; ^2^ Marine Evolutionary Ecology GEOMAR Helmholtz Centre for Ocean Research Kiel Kiel Germany

**Keywords:** microbe, microbiome, microbiota, pipefish, Syngnathidae

## Abstract

Bacteriophages play a crucial role in shaping microbial community dynamics in marine systems and have the potential to stimulate surges in pathogenic bacteria, facilitating disease outbreaks. Notwithstanding, bacteriophages also serve as valuable biocontrol agents, underscoring their huge potential for aquaculture therapy treatments. Empirical insights into the intricate tripartite interplay upon exposure to a virulent bacterium, its specific phages, and the host gut microbiome could improve our understanding of how bacteria–phage interactions behave in a natural microbial system. This investigation assessed the influence of a virulent *Vibrio alginolyticus* (K01M1) infection, in tandem with lytic (фSt2) and filamentous (фK04M1) *Vibrio*‐specific phages, on the broad‐nosed pipefish ( *Syngnathus typhle* ) gut microbiome using 16S rRNA amplicon sequencing and non‐intrusive gastric swabbing. The pipefish microbiome structure was not impacted by *Vibrio* and phage introductions, while the different infection regimes shaped *Vibrio*‐specific dynamics. In the filamentous phage and *Vibrio*‐only treatments, *V. alginolyticus* abundances spiked 12 h post‐ingestion. In contrast, *V. alginolyticus* numbers in the lytic phage and control treatment were significantly reduced, suggesting phage activity and specific elimination of the introduced bacteria. Assisted by relative true‐gut tissue samples, a newly implemented non‐intrusive swabbing method was successful at discerning the activity of two contrasting phages and supports previous work that encourages the use of фSt2 in bacteriophage treatments. Identifying *Vibrio*‐specific phages with similar positive characteristics could be beneficial for the aquaculture trade, which is currently heavily impacted by the antibiotic crisis.

## Graphical abstract

Utilising 16S rRNA amplicon sequencing and a novel non‐invasive swabbing technique, this investigation demonstrated that introduced bacteriophages can modulate the dynamics of the marine bacterium *Vibrio alginolyticus* within a tripartite system involving the gut microbiome of the pipefish, *Syngnathus typhle* , without disrupting core microbial communities. The swabbing method reliably detected differential phage activity, underscoring the therapeutic potential of lytic phages such as фSt2 for targeted pathogen control in aquaculture.

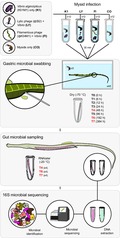

## 1 Introduction

Marine microorganisms have evolved along a continuum of symbiotic, commensal, opportunistic, and pathogenic states, with many capable of shifting activities in response to environmental changes (Wilson and Hastings 1998; Thompson et al. 2004). In turn, changing environments often dictate the co‐evolutionary outcomes of interacting species that exist within their realm of influence (Hendry and Kinnison 1999; Reusch and Wood 2007). Changes in the environment can influence pathogen fitness traits as indicated by increased replication rate (Mostowy and Engelstädter 2011; Wolinska and King 2009), contribute to immunologically compromised hosts (Wendling et al. 2013; Wendling and Wegner 2013), and consequently impact virulence evolution (Anderson and May 1982; Bull 1994; Ewald 1994; Ebert and Herre 1996; Frank 1996; Alizon et al. 2009; Engering et al. 2013). Biotic factors such as temperate bacteriophages can also contribute to increased pathogen virulence by supplementing bacteria with accessory genes (Waldor and Mekalanos 1996; Wagner and Waldor 2002; Austin et al. 2003; Payne et al. 2004). In contrast, some ‘phages’ have been shown to combat disease by lysing virulent disease agents, lending to the growing evidence that phage activities play an important role in manipulating the microbial environment and influencing the complex tripartite interactions between the host gut microbiome, bacteria and phage (Levin and Bull 2004; Rohwer and Thurber 2009; Harrison and Brockhurst 2017; Breitbart et al. 2018).

Phage lifecycles have evolved towards a number of subtle but distinguishable forms, each possessing unique characteristics and cellular repercussions. Lytic phages transmit horizontally by invading targeted bacteria, carrying out rapid phage replication and virion release following cell lysis, from which this lifecycle earns its name (Popescu et al. 2021; Chevallereau et al. 2022). Conversely, lysogenic phages are vertically transmitted without destroying the host cell, gradually integrating into the bacterial genome to form a ‘prophage’, which is then replicated following bacterial cell division (Weinbauer 2004). Temperate phages are capable of switching between these two lifecycles, while filamentous phages can release virions without cell lysis in a chronic process or adopt a lysogenic state if needs dictate (Rakonjac et al. 2011; Sausset et al. 2020).

Lytic phages attracted interest for their therapeutic potential in the early 20th century (Summers 2012; Wittebole et al. 2014), but the rise of antibiotics largely replaced phage therapy in the West. However, phage therapy has remained prominent in the East, especially in aquaculture, and is now being reconsidered in the West as an alternative to antibiotics, which can lead to adverse side effects such as resistant bacteria selection, gut microbiota disruptions and allergic reactions (Nakai et al. 1999; Sulakvelidze et al. 2001; Nakai and Park 2002; Merril et al. 2003; Defoirdt et al. 2011; Dethlefsen and Relman 2011; Ryan et al. 2011; Oliveira et al. 2012; Richards 2014; Blumenthal et al. 2019). Bacteriophage therapy, as it is known, represents an alternative, highly specific treatment against harmful ‘superbug’ bacteria that develop resistance to multiple antibiotics (Matsuzaki et al. 2005; Kortright et al. 2019). Identifying highly specific phage–bacteria interrelationships is often challenging; however, the specificity of phage–bacteria interactions can afford an advantage over non‐specific antibiotic treatments with fewer unwanted side effects to the detriment of the host microbiome (Nieth et al. 2015). Despite its medical potential, bacteriophage therapy is still comparatively under‐researched and would benefit from further systematic and fundamental in vivo studies that focus on pertinent virulent bacteria and their phage occupants.

*Vibrio* bacteria are the most abundant and diverse opportunistic bacteria in the marine realm, with some pathogenic phylotypes known to cause vibriosis in the natural environment (Blake et al. 1979; Thompson et al. 2004). These cases are often linked with seasonal salinity and temperature changes, with increased prevalence occurring during the warmer months (Martin et al. 2002; Dayma et al. 2015). Vibriosis is one of the most prevalent diseases affecting a wide range of crustacean, fish, and shellfish aquaculture practices, leading to significant economic losses and health concerns. Additionally, human exposure to contaminated aquatic environments, through ingestion or wounds, can result in severe infections (Baker‐Austin et al. 2018; Ina‐Salwany et al. 2019; Sanches‐Fernandes et al. 2022).

Bacteria‐phage studies are often carried out in vitro with liquid bacterial cultures with a single bacterial strain (Smith and Huggins 1983; Pavlova et al. 1997; Gupta and Prasad 2011; Cieplak et al. 2018). In addition, in vivo mammalian studies have used bacteriophage cocktails to assess the influence on bacterial targets, the immune system, and host–gut microbial communities (Maura et al. 2012; Febvre et al. 2019; Hsu et al. 2019). In fish, some aquaculture‐related experimental studies on the gut have explored the phage protective qualities (Jun et al. 2013; Christiansen et al. 2014; Wendling et al. 2017; Cafora et al. 2019), but few assessed the phage–bacteria interactions on the microbiome (Donati et al. 2022). All of the aforementioned studies used either extracted tissue or collected faecal samples to assess microbial communities, emphasising the need to develop an alternative, non‐intrusive methodology that aligns more closely with the 3 Rs principle (Russell and Burch 1959). Such a method would help enable targeted sampling at the primary site of phage–bacteria interactions in the gut while minimising the risk of contamination. From an experimental perspective, there also appears to be a clear absence of comparative research assessing the effects of distinct bacteriophages adopting alternate lifecycles in parallel within the fish gut. A better understanding of individual phage influences on the tripartite relationship in the fish gut could aid in designing optimal phage cocktails for targeting specific bacterial diseases in farmed fish while also providing insights into dynamics that may lead to crashes in wild fish stocks.

The broad‐nosed pipefish ( *Syngnathus typhle* ) is part of the enigmatic syngnathid fish group, renowned for their bizarre morphologies and their unique male pregnancy evolution (Stölting and Wilson 2007). Syngnathids have attracted interest due to their unusual immune repertoires, while other studies have advocated their use in phage–bacteria research (Wendling et al. 2017; Goehlich et al. 2019; Chibani, Hertel, et al. 2020; Chibani, Roth, et al. 2020; Roth et al. 2020; Parker et al. 2023). To investigate the influence of phage–bacteria interactions on the fish gut microbial composition over time, *S. typhle* was experimentally inoculated with either lytic or filamentous phages in combination with a known specific target, *Vibrio alginolyticus* . Repeated gastric swabbing and terminal gut tissue sampling in combination with 16S rRNA amplicon sequencing were used to assess the influence of the respective bacteriophages on the pipefish microbiome and *Vibrio* community over time.

In line with previous syngnathid studies that successfully isolated *Vibrio* from the gut (Wendling et al. 2017; Chibani et al. 2023), it was hypothesized that (i) the introduced *V. alginolyticus* (K01M1) strain would be detectable in treatments from 6 h post‐ingestion onwards using non‐intrusive gastric swabs. Successful phage therapy practices require high phage–bacteria interaction specificity (Ryan et al. 2011). Previous reports suggest the phages utilised in this study have a strong specific affinity for *V. alginolyticus* (Kalatzis et al. 2016; Wendling et al. 2017); therefore, it was hypothesised that (ii) all phage introduction treatments, independent of their lifestyle, would not negatively impact the relative abundance of other bacterial strains within the microbiome, suggesting a stable gut microbiome despite the introduction of phages. Based on the physiological characteristics of the two bacteriophages used in this investigation, we hypothesised that (iii) treatments with lytic phage introduction would result in a reduced *V. alginolyticus* relative abundance in comparison to the filamentous phage and *Vibrio*‐only treatments, while due to a less destructive lifecycle, (iv) the filamentous phage treatment would exhibit similar *V. alginolyticus* abundances to phage‐devoid treatments.

## 2 Materials and Methods

### 2.1 Ethics Statement

All research conducted in this investigation is in accordance with German animal welfare law and the ethical approval provided by the Ministerium für Energiewende, Landwirtschaft, Umwelt, Natur und Digitalisierung (MELUND) Schleswig–Holstein under the permit number: Ves.‐Nr.: V 242‐35168/2018 (63‐7/18). All fish used in this study were aquarium‐bred, and the species is not endangered.

### 2.2 The Eukaryotic Host

*S. typhle* originating from wild‐caught populations of the Baltic Sea was bred for several generations and reared at GEOMAR aquaria facilities in Kiel at 18°C. Before the experiments, fish were fed twice a day with a mixture of frozen and live mysids.

### 2.3 The Bacteria

*V. alginolyticus* is a prevalent gram‐negative bacterium commonly found in the Baltic Sea and other marine domains (Oberbeckmann et al. 2011; Böer et al. 2012). *V. alginolyticus* (K01M1) samples were revived from cryopreserved cultures previously isolated from nine healthy *S. typhle* individuals (Roth et al. 2012; Wendling et al. 2017). The K01M1 strain was selected for this investigation based on previous reports and laboratory trials confirming its selective vulnerability to infection by the two phage sub‐types used here (Wendling et al. 2017).

Isolate subsamples were preserved in 15 PSU liquid Medium101 (0.5% (w/v) peptone, 0.3% (w/v) meat extract, and 1.5% (w/v) NaCl and 25% glycerol in Milli‐Q water) and thawed gradually at 25°C while shaken at 180 rpm overnight. A day later, samples were diluted (1:10) in fresh medium and grown for a further 1.5 h (22°C at 180 rpm) to encourage exponential culture growth. Samples were subsequently centrifuged (8000 rpm at 12°C), supernatant was removed, and pellets were washed in 40 mL sterile seawater. Wash steps were repeated two times more prior to pellet resuspension and sample pooling in a total of 45 mL. Two dilution series (−1 to −7) were created in phosphate‐buffered saline (PBS), and 100 μL was incubated on 101_15PSU agar plates (22°C) for 16 h. Plate count numbers were as follows: 7 × 10^7^ (MW cfu/mL) and 3.5 × 10^7^ (cfu/500 μL), which were used for the inoculum in this experiment.

### 2.4 The Bacteriophages

One lytic and one filamentous bacteriophage were selected for this experiment based on their ability to specifically infect the K01M1 strain, as demonstrated in laboratory trials and a previous study (Wendling et al. 2017). The lytic phage (фSt2) was originally isolated from *V. alginolyticus* strain V1 around the coastal waters of Crete in Greece and obtained in liquid culture (2 × 10^9^ mL^−1^) from the University of Copenhagen, Denmark (Kalatzis et al. 2016). To prepare фSt2 samples, 2 × 1600 μL aliquots were centrifuged for 2 min (13,000 rpm), supernatants were filtered through 0.2 μm filters, and products were stored in Medium 101 in the fridge at 4°C. *V. alginolyticus* K01M1 overnight cultures were diluted (1:100) and incubated for 1.5 h at 22°C and consistently shaken at 180 rpm. For phage re‐cultivation, 4.5 mL of *V. alginolyticus* K01M1 bacterial culture was mixed with 500 μL of фSt2 and incubated for a further 4 h at 22°C while being shaken at 180 rpm. The supernatant was collected following 2 min centrifugation (13,000 rpm) then filtered again through a 0.2 μm filter and stored at 4°C. The final фSt2 concentration used for the experiment was 3 × 10^9^ pfu/mL.

The filamentous phage (фK04M1) was previously extracted from *V. alginolyticus* strain K04M1 (Wendling et al. 2017). A subsample of cryopreserved K04M1 *V. alginolyticus* bacteria culture in 15 PSU liquid Medium101 was thawed overnight at 25°C while being shaken at 180 rpm. After 26 h, bacteria were pelleted following centrifugation at 13000 rpm, and the supernatant was fed through a 0.2 μm filter to isolate the фK04M1 phage culture. A final concentration of 5 × 10^10^ pfu/mL was used for фK04M1 during the inoculation experiment.

### 2.5 Spot Assays

In line with a previous study, standard spot assays were carried out for both phage types to confirm phage activity on a plate overlaid with Baltic seawater with 0.4% agar and 200 μL with the susceptible K01M1 culture (Wendling et al. 2017). Plates were kept overnight (~20 h) before assessing the effectiveness of each phage by the presence (susceptible) or absence (resistant) of plaque formation.

### 2.6 Experimental Design

**FIGURE 1:**
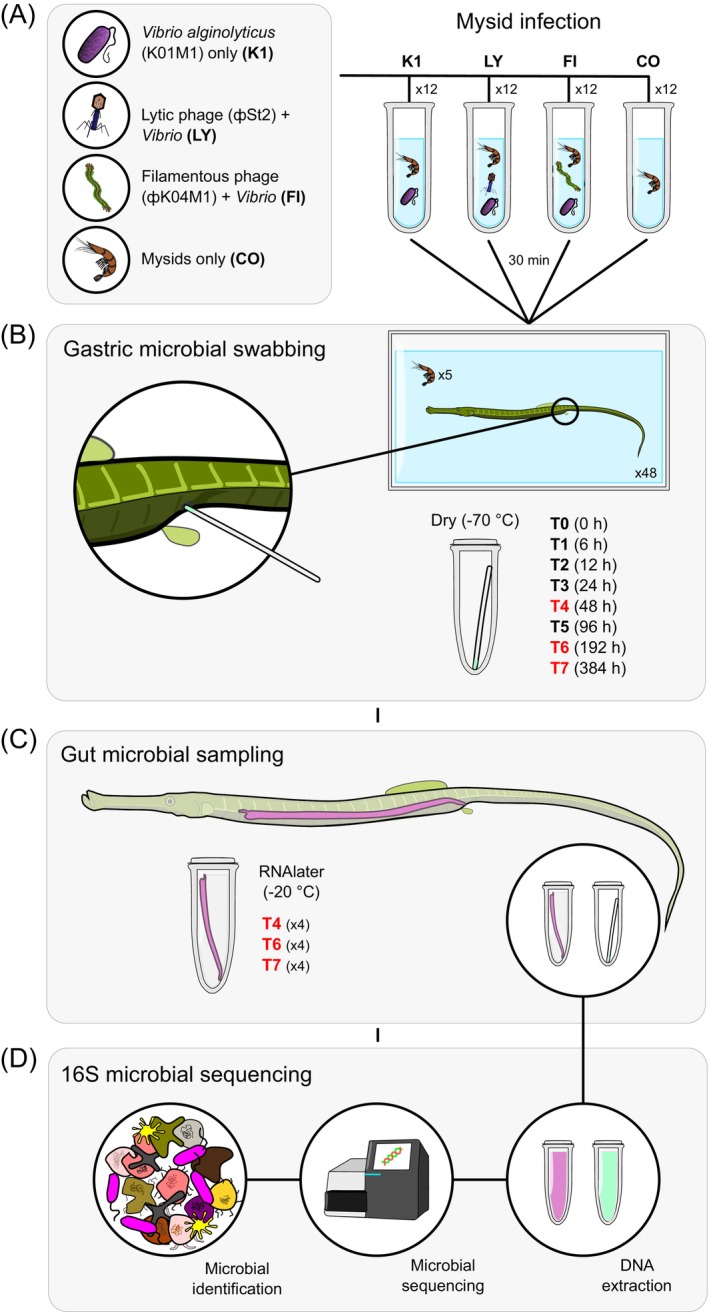
Refined schematic diagram depicting the following experimental steps used during this investigation: (A) Mysid incubation with bacteria and phage mixtures; (B) gastric microbial swabbing over a 16‐day period; (C) gut microbial sampling at three latter stages of the experiment and (D) 16S amplicon sequencing and microbiome assessment.

To understand the influence of lytic and filamentous phage cultures on the gut microbiota of *S. typhle* , a time series experiment was carried out that assessed how colonisation with *Vibrio* bacteria and bacteriophages, respectively, changed the host microbiomes over a period of 16 days. Live mysid cultures were incubated for 30 min with either *V. alginolyticus* K01M1 only (K1) (500 μL—K01M1 and 500 μL—Medium101 (15 PSU)), lytic phage фSt2 and K01M1 (LY) (500 μL—фSt2 and 500 μL—K01M1), filamentous phage фK04M1 and K01M1 (FI) (500 μL—фK04M1 and 500 μL—K01M1) or sterile seawater (CO) (500 μL—Medium101 and 500 μL—seawater) (Figure 1A) (Table S4).

Fish were placed in individual 20 L tanks and allowed to acclimate for 1 week in addition to being starved for 48 h prior to the experiment. Fish were fed once with five treated mysids added to each tank and consumed before sampling. Initially, 12 fish replicates were used per treatment group, resulting in 48 fish housed individually in 20 L tanks. Each tank system was isolated and maintained at 18°C for the duration of the experiment. Faeces were removed regularly to help maintain high water quality standards, while oxygen, pH, nitrite, and nitrate levels were monitored throughout the experimental period. To sample the microbial communities at the terminal end of the pipefish digestive tract, pre‐sterilised, absorbent paper swabs (ANTÆOS Absorbent Paper Points sterile, size 22 mm, 121 VDW GmbH) were used to swab the gastric pore/terminal gut opening of each fish (Figure 1B). Care was taken when handling fish out of the aquaria, and the sampling area was meticulously dried and disinfected using ethanol and DNA Away (Thermofisher Scientific) prior to swabbing to minimise environmental contamination. Gastric swabbing was carried out prior to mysid consumption (T0), and then after 6 h (T1), 12 h (T2), 24 h (T3), 48 h (T4), 96 h (T5), 192 h (T6) and 384 h (T7). For time points T0–T4, 12 swab replicates were used, while at time points T5, T6 and T7, there were 8, 8 and 4 swab replicates, respectively, per treatment. This replicate reduction was due to fish being euthanised for gut tissue sampling. All swabs were stored dry at −70°C in preparation for DNA extraction.

Gut tissue samples were dissected from four fish per treatment at time points T4, T6 and T7, resulting in a total of 12 fish per treatment over this period (Figure 1C). Consequently, the total number of swab samples at T5, T6 and T7 was reduced to 32, 32 and 16, respectively. Fish were euthanised with a lethal dose of MS‐222 (Tricaine, 500 mg L^−1^; Sigma‐Aldrich, Munich, Germany) before gut dissection, gut content removal, and gentle washing with PBS. Samples were initially placed in RNAlater at 4°C before relocation to −20°C until DNA extraction. To minimise the chances of contamination, all gut dissections were carried out under the sterile bench.

### 2.7 DNA Extraction, PCR Amplification and Sequencing

Thawed microbial swab tips and gut samples were homogenised using 180 μL of lysozyme solution (20 mM Tris CL pH 8.0, 2 mM sodium EDTA, 1.2% Triton X‐100, lysozyme 20 mg/mL) in lysing matrix A‐tubes (MP Biomedicals) using a tissue shredder (Qiagen, Hilden, Germany). For DNA extraction, the DNeasy PowerSoil Kit and standard protocol were used (Qiagen), in addition to an improved gram‐positive bacteria pre‐treatment used previously (Korsch et al. 2018; Tanger et al. 2024). DNA quality was then assessed using a NanoDrop‐1000 spectrophotometer (NanoDrop), and amplification of the V3–V4 hypervariable 16S rRNA gene region was targeted (341F‐805R), in line with a previously established protocol (Tanger et al. 2024). Library preparation was carried out at the Institute for Experimental Medicine (UKSH, Kiel), and paired‐end sequencing (2 × 300 bp) was conducted via Illumina MiSeq (Illumina, USA) at the Institute of Clinical Molecular Biology (IKMB) in Kiel (Figure 1D).

### 2.8 Data Analysis

Amplicon read sequences were demultiplexed before primer cutting, filtering, chimera removal, sequence quality checks, and denoising using DADA2 via QIIME 2 (v2022.8). By utilising the V3–V4 region as a reference, the Silva (v132) (Quast et al. 2012) was used for ribosomal rRNA alignment and taxonomic assignment. Chloroplast and mitochondrial‐associated 16S rRNA sequences were removed before continuing with downstream analyses.

Initial alpha rarefaction curves were used to confirm the sampling success of microbial communities and the observable clarification of species at various sequencing depths. Resulting operational taxonomic units (OTUs) were carried forward for analysis using Phyloseq (v1.42) (McMurdie and Holmes 2013) in R (v4.2.2) (R Core Team 2013). Sequence variants without a clear taxonomic class were removed prior to consolidating variants into ASV‐level groups. Taxa present in fewer than four samples and with less than two counts were filtered prior to converting the remaining taxa to relative abundance levels.

#### 2.8.1 Whole Microbiome Diversity Analysis

Local mean species diversity (α‐diversity) among microbiome strain constituents was assessed using Faith's phylogenetic diversity (Faith PD) and Shannon diversity and observable richness measures via the microbiome package for both swab and gut tissue‐derived data subsets (Lemos et al. 2011; Lahti and Shetty 2017). For α‐diversity data sets, linear mixed‐effect models were adopted to assess treatment, timepoint, and interaction effects. Repeated measures were accounted for using a fish individual (Aquarium no.) (Brooks et al. 2017; De Boeck et al. 2011; Kuznetsova et al. 2017). The implementation of repeated measures was not applied to the analysis of the gut tissue sample data set as it was derived from sacrificed individuals. For each model, ANOVA (type III) was used to assess overall treatment, time point and treatment: time point interaction effects.

As with the α‐diversity assessments, β‐diversity analyses (Bray–Curtis dissimilarity) were conducted separately for swab and gut tissue sample data sets. Treatment, time point and interaction effects were assessed using repeated measures PERMANOVA (data ~ treatment × time point) (999 permutations) with fish aquarium used as a block factor (strata = data$Aquarium).

#### 2.8.2 Indicator Species Analysis

Following β‐diversity assessments of the whole microbiome from swab samples, the indicspecies R package (v1.8.0) and multiplatt function (Cáceres and Legendre 2009) (999 permutations) were utilised for indicator species analyses, evaluating the influence of specific taxa on treatment and time‐point fixed effects.

#### 2.8.3 *Vibrio* sp. Diversity and Abundance Analysis

To evaluate differences in *Vibrio* β‐diversity (Bray–Curtis dissimilarity), a repeated measures PERMANOVA was conducted on a *Vibrio*‐only data set derived from the swab samples (data ~ treatment × time point) (999 permutations). Fish aquaria were included as a blocking factor (strata = data$Aquarium). To further assess any significant interaction effects, pairwise PERMANOVA was conducted to assess treatment differences at each time point with FDR correction applied for *p* value adjustment.

To assess differences in *Vibrio* relative abundance, a repeated measures linear mixed model was fitted (lmer(data ~ Treatment × Time point + (1 | Aquarium), data = Vibrio_abundance)), with treatment, time point and interaction effects being assessed further using ANOVA (type III). Optimal model performance was achieved by applying a Box–Cox transformation (Osborne 2010) to the *Vibrio* species data set beforehand.

#### 2.8.4 *V. alginolyticus* Abundance Analysis

To understand *V. alginolyticus* relative abundance differences, a repeated measures generalised linear mixed‐effects model (GLMM) was used on a *V. alginolyticus* data subset to evaluate treatment, time point and interaction effects (glmmTMB(Alginolyticus_abundance ~ Treat × Time + (1|Aquarium))). This model was chosen in accordance with the data's distribution, and ANOVA (type III) was used to assess overall treatment, time point and treatment: time point interaction effects. To assess significant interaction effects further, estimated marginal means were calculated for each model using the emmeans package (Lenth 2025), in preparation for pairwise comparisons of treatments at each time point, with Tukey correction applied for *p* value adjustment.

To assess the effectiveness of the gastric swabbing technique at determining accurate *Vibrio* community proportions, relative abundance swab data were correlated with the matching gut tissue samples extracted at time points T4, T6, and T7.

#### 2.8.5 Principal Component Analyses (PCAs)

PCA was performed on whole microbiome data subsets corresponding to each time point to visualise treatment‐related differences. Statistical assessments across PC1–4 were conducted using multivariate analysis of variance (MANOVA) for each time point representation. If significant differences were identified (MANOVA; *p* < 0.05), individual ANOVAs were used to identify the most influential principal components, on which post hoc Tukey tests were performed to extract treatment differences.

To assess differences in *Vibrio* composition across treatments, an exclusive *Vibrio* data subset was established, excluding all taxa not aligning with the *Vibrio* genus. As with the whole microbiome data sets, PCA was carried out for each time point, and MANOVA was conducted across PC1–4. Any significant time points were investigated further using ANOVA, and then pairwise treatment differences were evaluated using post hoc Tukey testing. *Vibrio* strain loading values were imprinted upon PCA visualisations that highlighted significant treatment disparities in order to help with the interpretation of treatment‐specific influences of each *Vibrio* strain. *Vibrio* strain deductions were based on NCBI nucleotide BLASTN+ reports (v2.16.0) (Altschul et al. 1997; Camacho et al. 2009), with the top 100 hits considered. As *V. alginolyticus* was introduced in this study, efforts were made to identify units with a high likelihood of a positive *V. alginolyticus* match. In turn, units with > 5 *V. alginolyticus* hits, 100% query coverage, and the highest max score were interpreted as *V. alginolyticus* . To support strain similarity deductions, a phylogenetic tree of the *Vibrio* DNA sequences was constructed using the TN93 (Tamura‐Nei, 93) model.

## 3 Results

### 3.1 Read Abundances

A total of ~21.8 M reads were attained from all swab, gut and water control samples (380 samples). Across 366 biological samples, a total of ~21.6 M raw reads were accrued following sequencing, with swab and gut samples accounting for ~18.2 M (~57,100 per replicate) and ~3.3 M (~71,000 per replicate) reads, respectively. Additional read statistics can be found in Tables S1–S3 and S5–S6.

### 3.2 Whole Microbiome Diversity

#### 3.2.1 α‐Diversity

To assess the general localised microbial diversity in this investigation, α‐diversity measures were adopted (Shannon diversity, observed richness and Faith's PD) (Table S7). All three α‐diversity assessments exhibited a significant time‐point fixed effect (*Shannon diversity*: *p =* 1.45 × 10^−13^; *Observed richness*: *p =* 1.07 × 10^−12^; *Faith's PD*: *p =* 2 × 10^−16^); however, no significant overall treatment or interaction effect was observed (Tables S8–S10).

All α‐diversity assessments for the gut sample data set showed no significant overall treatment and interaction effects (Figures S1 and S2; Tables S11–S13). The only observation of note was that Shannon diversity exhibited a significant time‐point effect (*p =* 0.03).

#### 3.2.2 β‐Diversity

For swab samples, comparative microbial β‐diversity (Bray–Curtis) assessments between treatments and time points were conducted. Non‐metric multidimensional scaling (NMDS) of the Bray–Curtis dissimilarity matrix for all relative abundance swab data exhibited a robust overlap of all treatments and disparities with the water and swab controls (Figure S3). After removing water and swab controls, β‐diversity assessments utilising constructed Bray–Curtis dissimilarity matrices were explored (Figure S4). PERMANOVA assessments showed significant differences for the main factors, treatment and timepoint, in isolation (Treatment: *F*_3,285_ = 3.62, *R* ^2^ = 0.03, *p =* 0.001; Time point: *F*_14,285_ = 7.6, *R* ^2^ = 0.15, *p =* 0.001), while the interaction between treatment and time was not found to be significant (Table S14A). In turn, post hoc indicator species analysis yielded taxa‐specific treatments, irrespective of timepoint, with the genus *Blfdi19* shown to be associated with CO, LY and K1 (Table S16). Other indicator taxa for the control treatment included genera *Babeliales* and *Bradymonadales*, while the lytic phage treatment showed an association with a *Planctomyocete, SM1A02*. The K1 treatment was identified with another *Planctomyocete (Pla4)* and *Neochlamydia*, while no treatment indicator taxa were matched with the filamentous phage treatment. For the gut samples, no significant effect was observed for treatment, time point or interaction (Table S15).

### 3.3 Total Vibrio Abundance and Diversity

**FIGURE 2:**
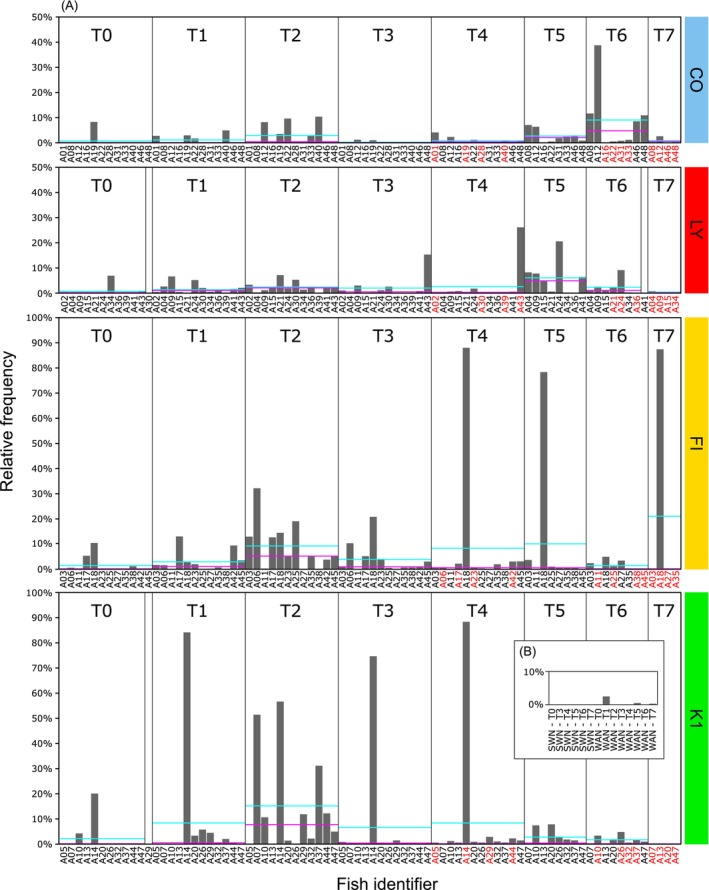
(A) *Vibrio* relative abundances for each time point in control (CO), lytic phage (LY), filamentous phage (FI) and *V. alginolyticus* (K1) gastric swab samples. (B) Swab (SWN) and water (WAN) negative controls indicated in cyan and pink lines indicate abundance mean and median values, respectively. Fish identifiers are derived from the unique resident tank identifier, and those highlighted in red at T4, T6 and T7 indicate fish used for gut tissue samples.

As *Vibrio* was introduced into the pipefish gut, the repercussive influence and comparative compositional characteristics of *Vibrio* communities were evaluated over the experimental period for all treatments. Swab relative abundance observations showed a *Vibrionaceae* spike at 12 h (T2) in each treatment (Figures S5 and S6). Further examination at the genus level highlights a large portion of these taxa as *Aliivibrio*, while a smaller subset represents *Vibrio*. *Vibrio* relative abundances were assessed for treatment, timepoints, and interaction effects using a fitted linear mixed effect model; however, the only significant effect was attributed to time point (*p* = 2 × 10^−16^) (Figure 2A,B) (Table S18).

To assess the *Vibrio* β‐diversity (Bray–Curtis) of swab samples, repeated measures of PERMANOVA were utilised. Results showed a significant treatment and time point effect (Treatment: *F*_3,220_ = 5.06, *R* ^2^ = 0.05, *p* = 0.001; Time point: *F*_7,220_ = 5.09, *R* ^2^ = 0.12, *p* = 0.001), while also showing a significant interaction effect (*F*_21,220_ = 1.11, *R* ^2^ = 0.08, *p* = 0.009), which encouraged further pairwise PERMANOVA comparison assessments (Table S17A). *Vibrio* β‐diversity was found to be significantly different in FI and K1 when compared to CO at time points T1 (FI: *F*_1,17_ = 4.28, *R* ^2^ = 0.2, *p* = 0.008, K1: *F*_1,17_ = 3.79, *R* ^2^ = 0.18, *p* = 0.008), T2 (FI: *F*_1,13_ = 5.61, *R* ^2^ = 0.3, *p* adj = 0.014, K1: *F*_1,14_ = 6.77, *R* ^2^ = 0.33, *p* = 0.008) and T4 (FI: *F*_1,18_ = 2.38, *R* ^2^ = 0.12, *p* = 0.032, K1: *F*_1,18_ = 4.31, *R* ^2^ = 0.19, *p* = 0.008). K1 *Vibrio* diversity was also found to be significantly different than in LY at T1 (*F*_1,20_ = 2.03, *R* ^2^ = 0.09, *p* = 0.028) and T2 (*F*_1,20_ = 4.58, *R* ^2^ = 0.19, *p* = 0.008), while at T2, FI and LY were shown to be significantly different (*F*_1,19_ = 3.41, *R* ^2^ = 0.15, *p* = 0.008) (Table S17B).

**FIGURE 3:**
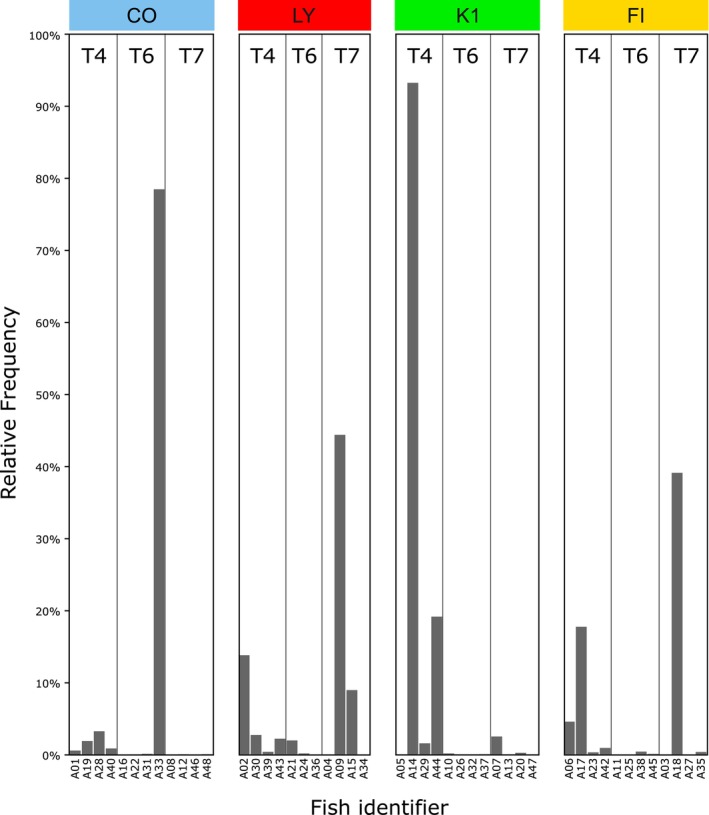
*Vibrio* relative abundances for each time point in control (CO), lytic phage (LY), filamentous phage (FI) and *V. alginolyticus* (K1) gut tissue samples. Fish identifiers are derived from the unique resident tank identifier.

For gut tissue samples, insufficient replicates with *Vibrio* populations rendered comparative statistical analysis between treatments unfeasible (Figure 3). However, a positive correlation was found between *Vibrio* relative abundances from gastric swabs and matching gut replicates (*R* ^2^ = 0.39, *p* = 1.6 × 10^−6^) (Figure S7 and Table S24).

### 3.4 *V. alginolyticus* Abundance

*V. alginolyticus* relative abundance differences following GLMM analysis revealed significant overall treatment (*p* = 0.0066), time point (*p* = 6.39 × 10^−8^) and interaction (*p* = 0.014) effects (Table S19). Estimated marginal means‐derived, pairwise comparisons were used post hoc to investigate the drivers of the interaction effect, with T2 showing significantly greater *V. alginolyticus* abundances at K1 (estimate = −2.2, SE = 0.36, *z* = −6.12, *p =* 5.6 × 10^−9^) and FI (estimate = −1.72, SE = 0.36, *z* = −4.7, *p =* 1.5 × 10^−5^) compared with CO (Table S20). A similar pattern was found when compared with LY at T2, with K1 (estimate = 1.99, SE = 0.35, *z* = 5.67, *p =* 8.8 × 10^−8^) and FI (estimate = 1.50, SE = 0.36, *z* = 4.21, *p =* 1.49 × 10^−4^) again showing a significantly higher *V. alginolyticus* abundance.

### 3.5 PCA

#### 3.5.1 Whole Microbiome

To provide an additional perspective on treatment effects at each time point, PCAs were conducted. Time‐point PCA plots (PC1:4) displayed a consistent sample overlap, with MANOVA assessments indicating no significant differences between the treatments across PC1 to PC4 (Figure S8 and Table S21A). The only significant difference observed when carrying out principal component ANOVA for each time point was PC1 of T3 (*p =* 0.03). In this instance, PC1 explained differences in the microbial communities of FI and LY replicates (Table S21B,C).

#### 3.5.2 *Vibrio* sp.

**FIGURE 4:**
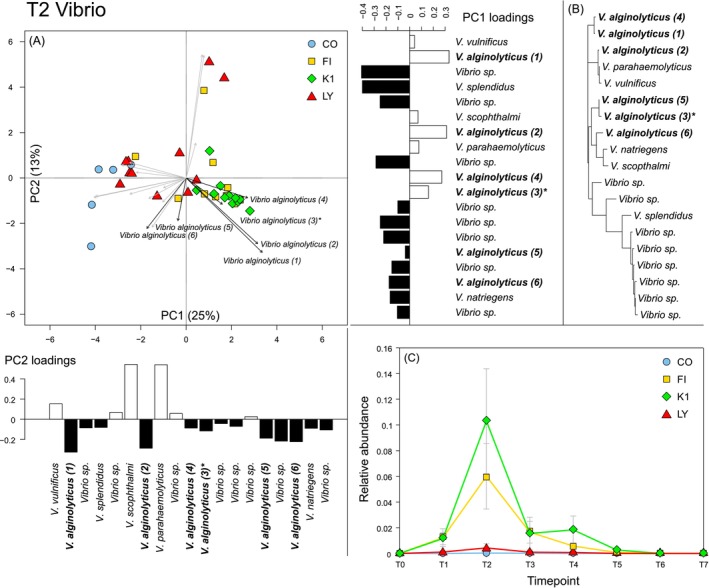
(A) PCA component plot of *Vibrio* strain data subset at T2 (12 h), with control (CO), filamentous phage (FI), *Vibrio* (K1) and lytic phage (LY) treatments. Loadings for all *Vibrio* strains influencing the spread of replicates are represented by dispersing arrows, with dark labelled arrows indicating *V. alginolyticus* strains and the introduced K01M1 strain (*). *Vibrio* strain loading values for PC1 and PC2 are also represented in the flanking bar charts, with positive and negative loadings signified in white and black, respectively. (B) Phylogenetic tree based on *Vibrio* strains influencing treatment differences at T2, created utilising the TN93 (Tamura‐Nei, 93) model. (C) Combined relative abundance of all *V. alginolyticus* over the course of the experiment.

Exclusive *Vibrio* PCA plots for each time point explain more variation with the formation of clusters evident at T2 in particular (Figure 4) (Figure S9). MANOVA assessments for each time point (PC1:4) showed significant treatment differences at T1 (*p =* 0.005), T2 (*p =* 0.0002), T4 (*p =* 0.049) and T5 (*p =* 0.04) (Table S22A). ANOVA assessments on T1 principal component scores showed PC1 and PC2 to harbour significant treatment differences (*p* = 0.02 and 0.03, respectively); post hoc Tukey tests indicated that the FI and CO pairwise comparison was significantly different (PC1) (Table S22B,C). Treatment differences were most distinct at T2, with PC1 explaining the majority of the difference following ANOVA testing (*p =* 1.1 × 10^−8^). Post hoc Tukey's tests further highlighted the existence of significant differences between the control (CO) and each of the other three treatments, FI (*p =* 1 × 10^−6^), K1 (*p =* 1.1 × 10^−8^) and LY (*p =* 0.003). Additionally, LY *Vibrio* communities were shown to be significantly different from K1 (*p =* 5.1 × 10^−5^) and FI (*p =* 0.005) (Table S22C).

*Vibrio* PCA loadings at T2 show 19 *Vibrio* strains with different modes of influence of the four treatments (Figures 4 and S5). Six units were identified as potential *V. alginolyticus* strains, which is supported by phylogenetic observations derived from a high sequence similarity among all six units (Figure 4B). Eight taxonomic units were identified to genus level (*Vibrio* sp.), and in the context of PC1 loadings, they were all positively associated with LY and CO replicates, but not FI and K1. One sequence in particular ( *V. alginolyticus* (3)) matched specifically with the introduced K01M1 strain used in this study. Taking into account the loading influence of the six *V. alginolyticus* strains for both PC1 and PC2, four units were positively associated with K1 and FI treatment replicates at T2. Combined *V. alginolyticus* relative abundances at each time point for each treatment showed a spike at T2, similar to that of the overall *Vibrio* assessments (Figure 4C).

### 3.6 Gut Samples

Gut samples taken at time points T4, T6 and T7 (*n* = 4) were intended to support microbial swab assessments. Gut time‐point PCA plots depicted a similar trend to swab communities, with all treatment replicates except some disparate K1 individuals at T4 (Figure S10). No significant differences were found between treatments following MANOVA at time points T4, T6 and T7, which is in line with the corresponding swab time‐point analyses (Table S23). Further details regarding sequencing statistics, testing and taxonomy specifics adopted in this investigation can be found in Tables S1–S3.

## 4 Discussion

Compared to investigations centred on dual interactions, the study of tripartite interactions between phages, bacteria, and the host gut microbiome is progressing with the development of more complex research questions, with the aim of elucidating potential pathogenic threats, mutualistic benefits, and the role of environmental factors (Wendling et al. 2017; Marchi et al. 2023). Considering the world's oceans, understanding tripartite interactions could be of particular importance, as temperature and salinity fluctuations are commonplace and have been shown to influence virulent bacterial blooms, impact aquaculture stocks, and highlight impending problems with the warming climate (Lipp et al. 2002; Kimes et al. 2012; Vezzulli et al. 2012; Baker‐Austin et al. 2013; Archer et al. 2023). Research on marine fish has deconstructed the three‐way relationship by assessing the phage–bacteria interactivity in vitro (Goehlich et al. 2021, 2024), while others have explored the dynamics of the triplicity through the lens of a eukaryotic host immune response and its benefits within the aquaculture trade (Nakai et al. 1999; Wendling et al. 2017). This investigation builds on these connections by exploring the impact on the host gut microbiome, a feature that has been comparatively under‐researched (Donati et al. 2022), while adopting a continuous non‐intrusive sampling method.

*S. typhle* gut microbial communities (16S rRNA) were assessed following the introduction of the opportunistic *V. alginolyticus* in tandem with *V. alginolyticus* ‐specific filamentous and lytic bacteriophages. The impact of the bacteriophage treatments on the host gut microbiome was investigated using comparative α‐ and β‐diversity metrics. Time was the only significant effect when assessing microbial α‐diversity, with no significant treatment or interaction effect observed. β‐diversity analyses highlighted a significant overall time point and treatment effect; however, importantly, they showed no significant interaction effect. In turn, only treatment‐specific indicator taxa were investigated further. Analyses revealed that control, lytic phage treatment and *Vibrio*‐only treatments shared a taxa known as *Blfdi19*, of which its role remains unknown but has been observed in the gut of the marine dwelling sea cucumber *Apostichopus japonicus* (Deng et al. 2024). The indicator taxa *Babeliales* and *Bradymonadales*, found in the control group, are typically associated with saline environments and marine sediments, respectively (Wang et al. 2015; Iqbal et al. 2021). However, their roles in the fish gut are not yet well understood. *Neochlamydia*, which was an indicator in the *Vibrio*‐only treatment, has also been detected in the gut of aquatic species previously (Mandal et al. 2024; Kanika et al. 2025). Finally, *SM1A02*, an indicator taxon in the lytic phage treatment, remains unclassified. Based on these findings, inferring specific fitness benefits or roles within the pipefish gut microbiome is challenging. Furthermore, without the context of the treatment‐time effect, interpretations of the potential treatment effects over the experimental period were not feasible. Therefore, aside from a single PCA result (24 h) that displayed a weak distinction between filamentous and lytic phage treatment communities, treatment comparisons at each time point highlighted no significant differences, suggesting that phage/*Vibrio* treatments did not alter the diversity of the host gut microbiome in this investigation. This is in line with several mammalian studies that have reported that high lytic phage doses do not stimulate a significant change in gut microbial diversity (Tanji et al. 2005; Golomidova et al. 2007; Mai et al. 2010; Grubb et al. 2020), while others emphasised that regular phage dosage can result in the opposite (Bao et al. 2018; Hsu et al. 2019; Lin et al. 2019). This observation in the context of phage therapy is a beneficial contrast to antibiotic use, which has been shown to disturb the host gut microbiome (Patangia et al. 2022; Fishbein et al. 2023).

In a force‐fed salmon experiment that attempted to understand the passage time differences between dry and soaked food pellets, 50% of the gastric tract was found to be emptied between 6‐ and 12‐h post‐feeding (Aas et al. 2017). Another salmon study used inert faeces markers to understand gastrointestinal evacuation times of different diets, finding that all three food remnants could be detected after 12–15 h (Storebakken et al. 1999). Similar passage times could be attributed to *S. typhle* in this investigation, as a *Vibrio* spike was observed at 12 h, likely indicating the passing of the ingested *Vibrio*‐laced food. *V. alginolyticus* in particular was highly represented at 12 h in filamentous phage and *Vibrio*‐only treatments, and the K01M1 strain was successfully identified, supporting the first hypothesis of this investigation (i). These results, in addition to positive correlation with gut tissue samples, endorse the use of gastric swabbing in this investigation as a cohesive, non‐intrusive approach to gut microbial assessments.

The lytic phage life cycle comprises bacterial infection, rapid intracellular replication and host cell destruction (Sulakvelidze et al. 2001; Weinbauer 2004; Abedon 2008). A conclusive, modular bacterial host‐range framework for bacteriophages has not been forthcoming, but it is generally understood that most phages are capable of infecting within a single bacterial genus (Ackermann and DuBow 1987; Weinbauer 2004; Flores et al. 2011), with a few exceptions (Paolozzi and Ghelardini 2006; Dekel‐Bird et al. 2015; Walsh et al. 2023). Species range within a single genus can vary greatly among phages (Kauffman et al. 2018), but there is evidence to suggest that phages are more likely to infect a bacterium of the same clade from which it was derived (Wendling et al. 2018). Lytic phages (фSt2) used in this experiment appear to have been operating specifically on the *V. alginolyticus* that were introduced collectively, with reduced relative abundances in the lytic treatment matching more with the control, which did not receive bacterial input. This could indicate that the phages introduced in the lytic treatment have lysed a high proportion of *V. alginolyticus* bacteria, in contrast to the filamentous phage and *Vibrio*‐only treatments, where in the latter no phages were introduced, while the filamentous phages rather resemble a chronic infection that does not lyse their bacterial hosts (Sausset et al. 2020). Strain specificity in the lytic фSt2 is also supported by the fact that total *Vibrio* relative abundances showed no significant difference between the lytic phage and the other treatments. Therefore, results suggest that an upturn in lytic activity and the converse reduction in targeted bacterial numbers by the 12 h mark has occurred, and lytic phage treatment replicates share a more similar growth pattern with the control, supporting the third hypothesis of this study (iii).

Filamentous phages operate at a slower pace than their lytic counterparts, gradually releasing intracellularly formed virions in a chronic process that can slow cell growth without inducing cell lysis (Sausset et al. 2020). PCA assessments at 12 h demonstrated the filamentous treatment to be very similar to the *Vibrio*‐only replicates in terms of its *Vibrio* population structure, strongly influenced by *V. alginolyticus* units. Interestingly, *V. alginolyticus* numbers in the filamentous treatment were significantly higher than in the control and the lytic treatment. This could be an indication that the filamentous phage used in this experiment is inhibiting *V. alginolyticus* cell growth but its activity is not as prolific as the lytic phage. At 12 h, the perceived earliest point of detection, the *Vibrio*‐only treatment exhibited a significantly higher relative abundance of *V. alginolyticus* compared to the control and lytic phage treatment. This is a coherent finding, as the *Vibrio*‐only treatment replicates were free from destructive phage activity. Moreover, K01M1's positive influence in distinguishing the filamentous phage/*Vibrio*‐only treatment pair from control/lytic phage highlights that incorporated filamentous phages are operating in a chronic, rather than lytic form, strongly supporting the third hypothesis of this study (iv).

A previous study found that the фSt2 lytic phage used in this investigation was able to successfully infect a range of *V. alginolyticus* strains, as well as one *V. parahaemolyticus* and one *V. harveyi* strain, demonstrating a broad host range for *Vibrio* bacterial strains (Kalatzis et al. 2016). Similarly, the filamentous фK04M1 has been shown to be an effective infector of *V. alginolyticus* , having been extracted from the same bacterial strain used in this investigation (Skliros et al. 2016; Wendling et al. 2017). In this regard, фK04M1 phages have been shown to preferentially infect within the *V. alginolyticus* clade from which they were derived (Wendling et al. 2018). It is difficult to conclusively determine whether other bacterial strains were affected by the phage treatments used in this investigation, as minor fluctuations in smaller bacterial communities can be influenced by a number of other factors (Dong and Gupta 2019; Barreto and Gordo 2023). However, based on the α‐ and β‐diversity assessments, as well as the lack of combined *Vibrio* abundance differences between treatments in this study, bacterial communities appear consistent in their fluctuations across all treatments. This indicates that, throughout the time course of the experiment, individual treatments likely did not impact the microbiome structure and therefore support the second hypothesis of this study (ii). It should be noted that a different outcome may have been reached if multiple treatment doses had been implemented, a trend that has been discussed previously (Bao et al. 2018; Hsu et al. 2019; Lin et al. 2019). The insurance of an uncompromised host microbiome following phage introduction is a requisite foundation of an effective phage therapy treatment (Kutter et al. 2015). Phage therapy has been carried out successfully in a number of aquatic species, including salmon, oysters and corals (Cohen et al. 2013; Higuera et al. 2013; Jun et al. 2014). In turn, the practice has huge potential, particularly for the aquaculture trade, which can rely too heavily on the use of antibiotic treatments (Romero et al. 2012). фSt2 has a number of related studies advocating its potential use in phage therapy treatments (Kalatzis et al. 2016, 2018; Skliros et al. 2016). While multiple dosing assessments and further validation would be beneficial to clarify фSt2 specificity and identify potential harmful side‐effects that can result from its use (Kalatzis et al. 2018), this investigation provides support for фSt2's use as a *V. alginolyticus* clade‐specific treatment.

This investigation raised a number of challenges and considerations that could be useful for future microbial experiments adopting a similar tripartite structure. The microbial results accrued using the swabbing method and the disparities with water/swab negative controls are promising, suggesting that the non‐intrusive method was free from environmental contamination. It is possible that the series of swabs was in part hindered by the reduction in sample size due to gut tissue sampling at 48, 192 and 384 h, rendering the results in this period more stochastic compared with the earlier time points. Gut samples were included in this investigation as a comparative supplement; however, there is a lack of contextual input with regard to the earlier stages. For this strategy to be effective, future investigations should include gut samples at the earlier stages and in numbers that do not compromise the number of swab replicates. Moreover, by significantly increasing the gut and swab replicate numbers across all time points, an even more rounded evaluation of the swab technique's effectiveness would be possible. On reflection, it stands to reason that retaining the swab replicate numbers throughout the experiment would have been more beneficial for understanding the latter gastric microbial communities. Based on the overall diversity fluctuations found across treatments in this experiment, the introduction of *V. alginolyticus* /phage cocktails did not evoke a detrimental or permanent change in the host–gut microbiome. Previous studies have reported that repeated phage doses can stimulate compositional shifts in the gut microbiome of mammals (Bao et al. 2018), which could also be the case in this study if repeated feedings were carried out. Future investigations should explore this point, as it could have implications for applied phage therapy treatments.

This study highlights subtle effect differences between lytic and filamentous phages in a host gut environment, with results suggesting that single‐dose phage treatments reduced *V. alginolyticus* abundance, without permanently impacting the microbiome structure over the course of the experiment. In addition, this is the first investigation to assess фSt2 within the pipefish gut, providing novel insights into host microbiome stability following phage introduction. Furthermore, this study established a novel gastric swabbing technique capable of supporting continuous and non‐intrusive experimental designs. фSt2's lytic activity is in line with previous claims for its potential use in *Vibrio*‐specific bacteriophage therapy, which could serve as an alternative or supplement to antibiotic treatments in industrial aquaculture.

## Author Contributions

**Jamie Parker:** data curation, formal analysis, writing – review and editing, writing – original draft, investigation, visualization. **Silke‐Mareike Marten:** methodology, writing – review and editing. **Jelena Rajkov:** methodology, writing – review and editing. **Franziska I. Theising:** methodology, writing – review and editing. **Arseny Dubin:** data curation, formal analysis, writing – review and editing. **Olivia Roth:** conceptualization, funding acquisition, methodology, supervision, writing – review and editing, investigation, visualization.

## Conflicts of Interest

The authors declare no conflicts of interest.

## Supporting information


**Data S1.** Supporting Information Figures.

**Data S2.** Supporting Information Tables.

## Data Availability

Raw sequencing data are available in NCBI SRA under project PRJNA1186123 at www.ncbi.nlm.nih.gov/sra/PRJNA1186123.
